# From Germline Susceptibility to Therapeutic Vulnerability: DNA Damage Response Gene Mutations Driving Multiple Myeloma Evolution and Precision Therapy

**DOI:** 10.1155/humu/7757115

**Published:** 2026-06-17

**Authors:** Qian Shen, Ying Wang, Liuhuan Cai, Juan Qian

**Affiliations:** ^1^ Department of Hematology and Lymphoma, Tumor Hospital Affiliated to Nantong University, Nantong, China, ntzlyy.cn

**Keywords:** DNA damage response, gene mutation, genomic instability, germline mutation, multiple myeloma, precision medicine

## Abstract

Multiple myeloma (MM) is characterized by genomic instability and therapeutic resistance. Emerging evidence indicates that germline DNA damage response (DDR) mutations, including BRCA1/2, ATM, and CHEK2 variants, contribute to MM susceptibility, clonal evolution, and treatment response. Inherited DDR defects promote chromosomal instability, reshape the immune microenvironment, and facilitate therapy‐driven disease progression. Recent advances in multiomics profiling, single‐cell sequencing, and liquid biopsy have improved the functional interpretation and clinical monitoring of DDR alterations. Moreover, DDR‐associated vulnerabilities provide opportunities for precision therapies, including PARP inhibitor–based synthetic lethality strategies. This review summarizes the mechanistic and clinical significance of germline DDR alterations in MM and highlights their translational potential in precision oncology.

## 1. Introduction

Multiple myeloma (MM) is a clonal plasma cell malignancy characterized by marked clinical heterogeneity and persistent therapeutic challenges, including high relapse rates, frequent drug resistance, and substantial variability in patient outcomes [1]. Although the incorporation of immunomodulatory drugs (IMiDs), proteasome inhibitors, and monoclonal antibodies has significantly improved survival over the past two decades, MM remains largely incurable. Most patients eventually develop relapsed or refractory disease, which underscores the urgent need to better understand the molecular determinants of disease progression and therapeutic resistance [2]. Genomic instability (GIN) is a central hallmark of MM and a major driver of its biological diversity and clinical behavior [1]. The MM genome is highly complex, encompassing numerical chromosomal abnormalities such as hyperdiploidy, recurrent immunoglobulin heavy chain (IGH) translocations, including t(4; 14) and t(11; 14), copy number alterations such as 1q gain and del(17p), as well as complex structural rearrangements including chromothripsis and chromoplexy [3, 4]. Approximately half of MM cases are hyperdiploid, characterized by trisomies of odd‐numbered chromosomes, whereas nonhyperdiploid cases more frequently harbor IGH translocations [4]. In addition, a subset of patients exhibits elevated mutational burden and enrichment of APOBEC‐associated mutational signatures [5]. Importantly, GIN in MM is a dynamic process rather than a static state. Under therapeutic pressure, subclonal populations undergo selection and expansion, leading to the emergence of high‐risk clones at the time of relapse [6]. The complexity of MM pathobiology is further increased by noncoding RNAs, such as the long noncoding RNA MALAT1, which has been implicated in regulating genomic stability and DNA damage response (DDR) pathways [7]. Within this framework, aberrations in the DDR system serve as a critical link between GIN and MM progression [8]. DDR defects arise from both inherited and acquired genetic alterations. Germline mutations in key DDR genes, including BRCA1/2, ATM, and CHEK2, are enriched in familial and early‐onset MM, contributing to baseline GIN [9, 10]. During disease evolution, somatic alterations such as biallelic inactivation of TP53 and loss of ATM further exacerbate GIN and promote clonal selection [2, 4, 5]. Although these defects facilitate tumor progression and therapy resistance, they also create potential therapeutic vulnerabilities. In this review, we provide a comprehensive overview of DDR alterations in MM, focusing on their roles in genetic susceptibility, clonal evolution, molecular pathogenesis, and prognostic stratification, as well as emerging targeted therapeutic strategies.

## 2. The Dual Physiological and Pathological Roles of the DDR System

**Figure 1:**
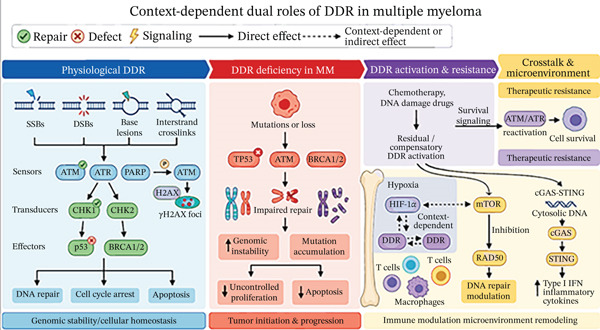
Context‐dependent dual roles of the DNA damage response in MM. The DDR maintains genomic stability via coordinated DNA repair and cell cycle regulation. In MM, mutations in key DDR genes impair repair capacity, driving genomic instability and progression. Crucially, the DDR exhibits a context‐dependent duality: Although deficiency promotes tumor initiation, residual or compensatory DDR activity under genotoxic stress supports cell survival and therapeutic resistance. Additionally, DDR signaling crosstalks with hypoxia pathways and the cGAS‐STING axis to reshape the bone marrow microenvironment.

The DDR system is a critical mechanism for maintaining genomic integrity by detecting and repairing various types of DNA lesions, including single‐strand breaks (SSBs), double‐strand breaks (DSBs), base modifications, and interstrand crosslinks [11]. This network is composed of damage sensors, signal transducers, and downstream effectors. Together, these components regulate cell cycle checkpoints, DNA repair, and apoptosis to preserve cellular homeostasis [12]. For example, ATM phosphorylates H2AX to generate *γ*H2AX foci, recruiting repair proteins to DSB sites [13]. Dysregulation of DDR, through gene mutations or aberrant expression, compromises genomic stability and drives tumorigenesis [11]. In MM, frequent alterations in key DDR genes, such as TP53, ATM, and BRCA1/2, impair DNA repair capacity and promote malignant phenotypes by allowing damaged cells to bypass apoptosis and sustain uncontrolled proliferation [8, 14]. Importantly, the DDR system exhibits a context‐dependent duality in MM (Figure 1). Although DDR deficiency promotes GIN and tumor initiation, residual or compensatory DDR activity can protect tumor cells under genotoxic stress, thereby contributing to therapeutic resistance [15]. Beyond its primary role in DNA repair, DDR signaling interacts extensively with other cellular pathways. For instance, hypoxia‐inducible factor 1*α* (HIF‐1*α*) has been reported to modulate DDR signaling under hypoxic conditions; however, its net effect may be context‐dependent, with some evidence suggesting it impairs rather than enhances DNA repair. Additionally, mTOR inhibition has been shown to influence repair processes by regulating RAD50 expression [15, 16]. DDR defects may also modulate the bone marrow microenvironment; cytosolic DNA accumulation can activate the cGAS‐STING pathway, triggering immune responses that impact MM progression [17]. Together, these findings underscore the complex and context‐dependent roles of DDR in MM and provide a strong rationale for the development of DDR‐targeted therapeutic strategies.

## 3. Landscape of DDR Alterations in MM: From Germline Susceptibility to Somatic Evolution

### 3.1. Germline DDR Variants and Genetic Susceptibility

Recent studies have highlighted a significant contribution of germline DDR gene variants to MM susceptibility [9, 10]. The prevalence of germline DDR mutations is notably higher in familial MM compared with sporadic cases (18% vs. 9.1%) [9]. Among these, BRCA1 and BRCA2 are the most frequently enriched genes, with strong associations with MM risk (BRCA1 OR = 3.9; BRCA2 OR = 7.0) [10]. Importantly, evidence of tumor‐specific loss of heterozygosity (LOH) in BRCA2 (observed in 5/8 cases) suggests a functional “second hit,” leading to complete loss of gene function [10]. Other DDR genes, including ATM and CHEK2, also contribute to inherited susceptibility, with reported carrier frequencies of 2.5% and 1.8%, respectively, in familial MM [9]. These germline alterations are not only associated with increased disease risk but also with earlier disease onset, with BRCA2 mutation carriers presenting 5–10 years earlier than noncarriers [10]. Moreover, approximately 30% of patients harboring germline DDR mutations have a family history of other malignancies, particularly breast and ovarian cancers [9]. Germline DDR status may also carry clinical implications. Preliminary evidence suggests that BRCA2‐mutated patients may experience improved progression‐free survival (PFS) following high‐dose melphalan and autologous stem cell transplantation (HR = 0.45; 95% CI: 0.22–0.92), though this finding requires validation in larger cohorts [10]. Collectively, these findings suggest that germline DDR defects establish a permissive genomic background that primes MM initiation.

### 3.2. Somatic DDR Alterations and Clonal Evolution

During MM progression, somatic alterations in DDR genes accumulate and play a central role in clonal evolution [4, 5]. Large‐scale genomic studies, including the CoMMpass dataset, have demonstrated that DDR mutations are widespread and dynamically enriched during disease progression and relapse [5]. Among these, TP53 alterations are particularly prominent, with biallelic inactivation (mutation combined with deletion or copy‐neutral LOH) increasing from approximately 5%–8% at diagnosis to 25%–30% in relapsed/refractory MM [2, 6, 18]. Similarly, ATM alterations occur in approximately 5%–10% of patients and are associated with poor prognosis [8]. MM evolution follows a branching pattern, in which subclones harboring additional DDR defects gain selective advantage under therapeutic pressure [6]. Single‐cell analyses reveal that up to 91% of patients harbor multiple subclones, with a substantial fraction already containing high‐risk DDR lesions at diagnosis [6]. These subclones can expand during treatment, exemplified by the marked increase in 1q gain clones from 16% at diagnosis to 92% at relapse [6]. Importantly, DDR alterations are also linked to treatment resistance, as TP53‐mutated MM exhibits inferior responses to proteasome inhibitors and IMiDs [2]. Thus, somatic DDR alterations act as key engines of clonal diversification and therapeutic escape. Consequently, somatic DDR alterations act as key engines of clonal diversification and therapeutic escape within a dynamic evolutionary framework that connects germline susceptibility to therapy‐driven selection.

### 3.3. The Two‐Hit Model and Structural Complexity

The “two‐hit” model provides a unifying framework for understanding DDR inactivation in MM, integrating germline predisposition with somatic events such as LOH [9, 10]. This mechanism has been well illustrated in BRCA2, ATM, and CHEK2, where germline mutations are frequently followed by somatic loss of the remaining allele [9, 10]. In parallel, MM is characterized by a high prevalence of complex structural variations, including chromothripsis and chromoplexy, which are closely linked to DDR deficiency. Chromothripsis occurs in approximately 10%–15% of MM cases and shows strong co‐occurrence with high‐risk lesions such as TP53 mutations and del(17p) [3]. Notably, patients harboring both DDR defects and complex structural rearrangements exhibit markedly inferior outcomes, reflecting synergistic effects on GIN [2]. Taken together, these findings support a convergent model in which germline susceptibility, somatic evolution, and structural genomic chaos collectively drive MM progression. Importantly, this integrated view highlights DDR deficiency not merely as a passive consequence of tumor evolution, but as an active and actionable vulnerability that may be therapeutically exploited.

## 4. Molecular and Microenvironmental Mechanisms by Which DDR Defects Drive MM Progression

### 4.1. Genomic Instability and Accelerated Subclonal Evolution

Defects in the DDR represent a central driver of genomic instability in MM, promoting the accumulation of tumor mutational burden (TMB) and fueling clonal evolution [1, 4]. Dysfunction of multiple repair pathways, including base excision repair (BER), nucleotide excision repair (NER), and homologous recombination (HR), contributes to elevated mutation rates [5]. Indeed, MM patients harbor significantly higher TMB than healthy individuals, with DDR‐deficient cases exhibiting a 2–3‐fold increase compared with wild‐type counterparts [5]. For example, some DDR‐deficient MM cases, particularly those with APOBEC‐associated mutational signatures, exhibit elevated TMB, although a direct quantification of TMB specifically in BRCA2‐mutated MM remains limited [19, 20]. This mutational diversity provides a substrate for Darwinian selection under therapeutic pressure, enabling the emergence of resistant subclones [6]. Longitudinal and single‐cell analyses demonstrate that minor subclones present at diagnosis can expand during relapse, as exemplified by the increase of 1q gain clones from 16% to 92% [6]. In parallel, DDR defects promote chromosomal instability (CIN), leading to aneuploidy and structural rearrangements such as chromothripsis, observed in 10%–15% of MM cases [3, 4]. These processes collectively accelerate clonal diversification and are strongly associated with treatment resistance, particularly in TP53‐mutant disease [2]. Thus, DDR deficiency acts as a fundamental engine driving both genetic diversity and evolutionary adaptability in MM.

### 4.2. Evasion of Apoptosis and Cell Cycle Dysregulation

DDR alterations enable MM cells to evade apoptosis and bypass cell cycle checkpoints, primarily through disruption of the TP53/MDM2 axis [14, 15]. Under physiological conditions, TP53 is activated in response to DNA damage to induce cell cycle arrest or apoptosis [14]. TP53 mutations or deletions are identified in approximately 10% of newly diagnosed MM cases and increase to nearly 30% in relapsed disease [2]. These alterations abolish TP53‐mediated protective responses and allow malignant plasma cells to survive despite persistent genomic injury. In parallel, overexpression of MDM2 further suppresses TP53 signaling by promoting TP53 ubiquitination and proteasomal degradation, thereby reinforcing apoptotic resistance and tumor cell persistence [15]. DDR defects also impair checkpoint signaling pathways; for instance, ATM mutations compromise CHK2 activation, leading to defective G2/M arrest and continued proliferation despite genomic damage [13]. In parallel, alterations in apoptotic regulators such as the BCL‐2 family further enhance cell survival, with increased BCL‐2 and reduced BAX expression observed in TP53‐deficient MM [14]. Collectively, these alterations uncouple DNA damage sensing from downstream cell fate decisions. This allows MM cells to survive and proliferate despite accumulating genomic lesions—a mechanistic rewiring that not only drives disease progression but also contributes to resistance against proteasome inhibitors and other standard therapies.

### 4.3. Crosstalk Between DDR Defects and the Bone Marrow Microenvironment

**Figure 2:**
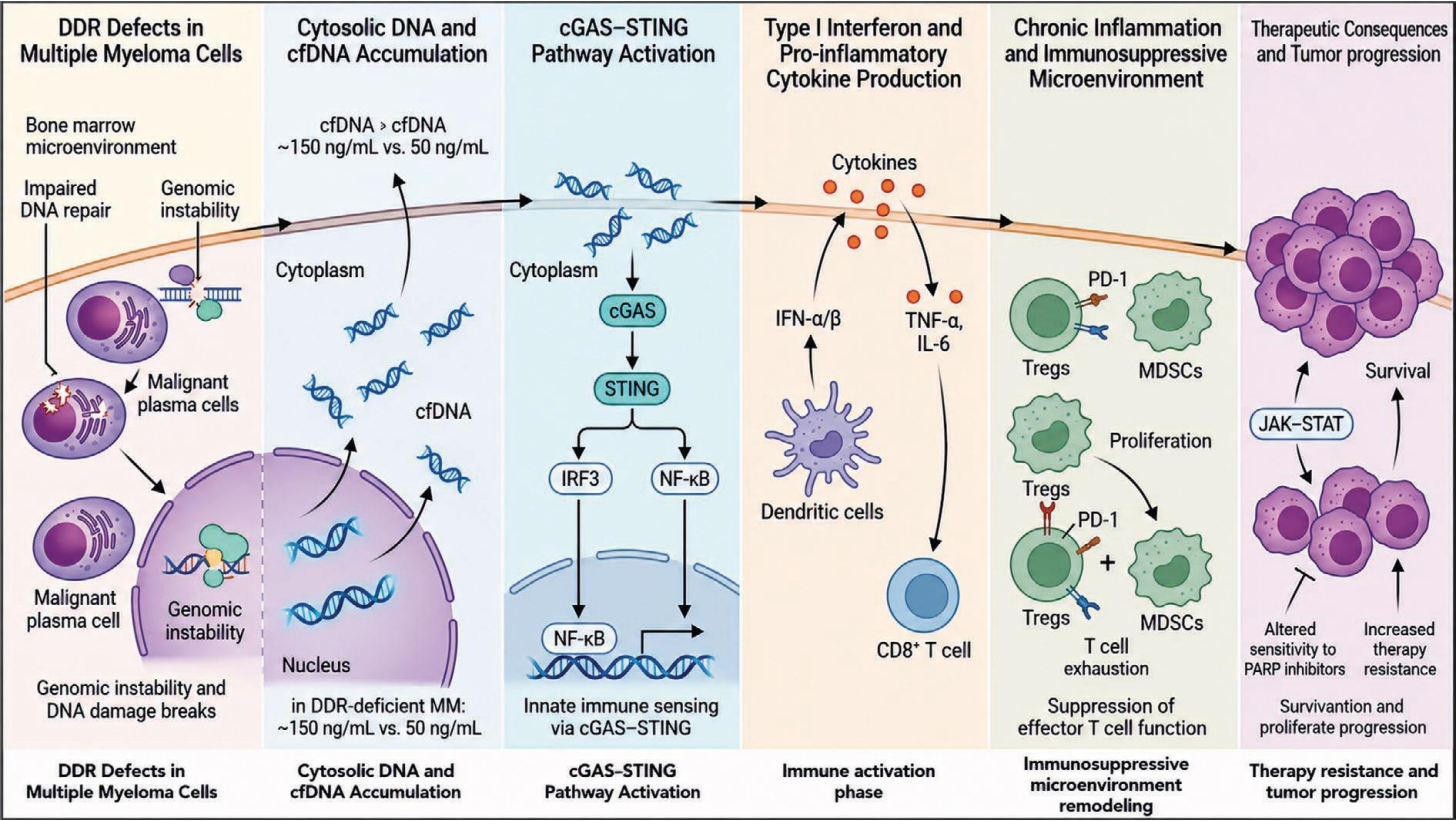
Mechanisms of immune microenvironment remodeling driven by DDR defects in MM. DDR defects lead to the accumulation of cytosolic DNA and cfDNA, thereby activating the cGAS–STING signaling pathway and inducing the release of Type I interferons and proinflammatory cytokines. This transiently enhances immune surveillance and promotes T cell recruitment. Subsequently, chronic inflammation drives the establishment of an immunosuppressive microenvironment, characterized by the expansion of Tregs and MDSCs, as well as T cell exhaustion. Ultimately, activation of signaling pathways such as JAK–STAT promotes tumor progression and contributes to therapeutic resistance.

The importance of the tumor immune microenvironment has been increasingly recognized across many cancers [21–23]. In MM, the bone marrow microenvironment similarly plays a critical role in shaping disease progression and therapeutic response. Beyond intrinsic tumor cell effects, DDR defects reshape the bone marrow microenvironment through the accumulation of cytosolic DNA and activation of the cGAS‐STING pathway [17, 24]. Impaired DNA repair leads to elevated levels of cytosolic cell‐free DNA (cfDNA), which activates cGAS and triggers STING‐mediated signaling. This process results in the sustained production of Type I interferons (IFNs) and proinflammatory cytokines [17]. Clinically, elevated cfDNA levels have been observed in DDR‐deficient MM patients (median 150 ng/mL vs. 50 ng/mL), accompanied by increased IFN‐*α* and TNF‐*α* expression [24]. Although this response may initially enhance immune surveillance, chronic activation paradoxically promotes an immunosuppressive microenvironment by expanding regulatory T cells and myeloid‐derived suppressor cells [17]. These changes impair effector T‐cell function and contribute to immune exhaustion. Furthermore, DDR–microenvironment interactions may influence therapeutic responses. For example, enhanced inflammatory signaling in DDR‐deficient contexts may exacerbate toxicity or modulate sensitivity to PARP inhibitors [19]. In addition, cytokine‐mediated activation of pathways such as JAK–STAT may directly support MM cell survival and proliferation [24]. Overall, DDR defects not only drive intrinsic tumor evolution but also orchestrate a permissive and immunosuppressive microenvironment, underscoring their multifaceted role in MM pathogenesis and therapy resistance (Figure 2).

## 5. Clinical Implications of DDR Alterations in Diagnosis and Risk Stratification

### 5.1. Prognostic Biomarkers and Risk Stratification Systems

The identification of reliable biomarkers is critical for precision oncology, as demonstrated across various malignancies where molecular signatures have guided targeted therapies and treatment decisions. In MM, DDR gene alterations serve as such biomarkers, providing a rationale for exploring PARP inhibitors through synthetic lethality strategies. Alterations in DDR genes, particularly TP53 mutation accompanied by deletion, represent one of the most powerful adverse prognostic factors in MM and can significantly refine current risk stratification models such as the Revised International Staging System (R‐ISS) [2, 10]. The prevalence of TP53 biallelic inactivation is approximately 5%–8% at diagnosis and increases to 25%–30% in relapsed disease [2]. Clinically, these alterations are associated with markedly inferior outcomes, with median overall survival (OS) of 12–18 months compared with 50–60 months in wild‐type patients, and significantly shorter PFS (HR: 2.5–3.0) [2]. Beyond TP53, other DDR genes, including ATM and BRCA2, are also linked to poor prognosis, with median OS of approximately 24 and 30 months, respectively [10]. Notably, DDR alterations provide prognostic information independent of traditional factors like ISS stage and lactate dehydrogenase (LDH) levels. Integrating DDR status into the R‐ISS framework enables more refined risk discrimination, identifying “ultrahigh‐risk” subgroups with extremely poor survival [2]. Collectively, these findings support DDR alterations as robust biomarkers that bridge molecular pathology and clinical risk stratification.

### 5.2. Multiomics‐Based DDR Prognostic Models

Multiomics‐based prognostic models have been increasingly applied across various cancers to improve risk stratification and guide personalized treatment [25–27]. The integration of multiomics data has enabled the development of DDR‐based prognostic models for personalized risk prediction in MM [28, 29]. These models incorporate genomic (mutations and copy number alterations), transcriptomic (gene expression), and epigenetic features to generate composite risk scores [28]. For example, a LASSO‐Cox model derived from 102 DDR‐related genes identified a five‐gene signature (DGCR8, POM121, TAF9, UPF3B, and BCAP31) that stratifies patients into high‐ and low‐risk groups with significantly different survival outcomes (median OS: 24 vs. 60 months; HR = 2.5) [28]. Similarly, transcriptome‐based signatures comprising multiple DDR genes demonstrate strong predictive performance, with concordance indices (C‐index) of 0.75–0.80 across independent cohorts [29]. Notably, integrative models combining genomic and transcriptomic data further improve predictive accuracy (C‐index up to 0.85) [28]. Beyond prognostication, these models may also inform therapeutic decision‐making, as high‐risk DDR signatures have been associated with increased sensitivity to PARP inhibitors [19]. Thus, multiomics‐based DDR models represent a critical step toward data‐driven precision medicine in MM.

### 5.3. Liquid Biopsy and Dynamic Monitoring

Liquid biopsy has emerged as a minimally invasive approach for real‐time monitoring of MM, with particular relevance for tracking DDR alterations and clonal dynamics [30, 31]. Circulating tumor DNA (ctDNA) serves as a key biomarker, showing high concordance (~90%) with bone marrow–derived genomic profiles for DDR mutations such as TP53 and ATM [30]. Moreover, ctDNA levels correlate with tumor burden and can achieve high sensitivity for minimal residual disease (MRD) detection [30]. Dynamic changes in variant allele frequency (VAF) of DDR mutations provide valuable insights into treatment response and disease progression. For instance, reduction of VAF below 0.1% is associated with prolonged PFS (50 vs. 20 months) [30]. Importantly, ctDNA analysis enables early detection of relapse, often preceding clinical progression by 3–6 months [31]. In addition, liquid biopsy can capture emerging resistance mechanisms, such as BRCA reversion mutations following PARP inhibitor therapy [31]. Overall, liquid biopsy–based monitoring of DDR alterations offers a powerful tool for longitudinal disease assessment, enabling early intervention and adaptive therapeutic strategies in MM.

## 6. Synthetic Lethality and Combination Therapeutic Strategies Targeting DDR Defects

### 6.1. PARP Inhibitors and Synthetic Lethality

PARP inhibitors exploit synthetic lethality in MM cells harboring homologous recombination deficiency (HRD), including BRCA‐like phenotypes and ATM alterations [19, 32]. Mechanistically, PARP inhibition impairs SSB repair, resulting in the accumulation of DNA DSBs that cannot be effectively resolved in HR‐deficient cells, ultimately leading to genomic instability and cell death [19]. Preclinical studies have demonstrated pronounced sensitivity of HRD MM cells to PARP inhibition; notably, BRCA2‐mutant MM cell lines exhibit substantially lower IC50 values for olaparib (0.5–1.0 *μ*M) compared with wild‐type counterparts (> 10 *μ*M) [19]. Early‐phase clinical trials further support this therapeutic vulnerability, reporting objective response rates (ORRs) of approximately 33% in relapsed/refractory MM, increasing to nearly 50% among patients harboring BRCA2 mutations [19]. Likewise, niraparib has shown promising activity in ATM‐mutant MM, with ORRs of approximately 25% [32]. Importantly, although therapeutic responsiveness is strongly associated with HRD status, as defined by genomic scar–based signatures, multiple layers of intrinsic and acquired resistance remain major barriers to durable clinical benefit [19, 31]. Collectively, these findings underscore the clinical limitation of PARP inhibitor monotherapy in MM and highlight the necessity for rational combination strategies aimed at disrupting tumor microenvironment–mediated protection, suppressing compensatory DNA repair pathways, and overcoming subclonal resistance mechanisms.

### 6.2. Interaction With Conventional Antimyeloma Therapies

DDR status critically modulates the response of MM cells to conventional therapies [2, 15]. Alkylating agents such as melphalan induce DNA crosslinks that require functional HR for repair. Accordingly, BRCA2‐deficient MM exhibits enhanced sensitivity to melphalan (response rates ~60% vs. 30% in wild‐type) due to impaired repair capacity [10]. In contrast, ATM alterations may confer variable responses, reflecting compensatory activation of alternative repair pathways such as NHEJ [2]. Proteasome inhibitors, including bortezomib, exert antimyeloma activity primarily through the induction of proteotoxic stress and apoptosis; however, their therapeutic efficacy is markedly attenuated in TP53‐mutant MM, with response rates of approximately 20% compared with nearly 60% in TP53‐wild‐type disease [2]. Mechanistically, loss of TP53 impairs apoptosis, whereas ATM alterations may activate prosurvival pathways such as NF‐*κ*B, further contributing to resistance [15]. These observations highlight DDR status as a key determinant of therapeutic vulnerability and underscore the importance of integrating molecular profiling into treatment selection.

### 6.3. Rational Design of DDR‐Based Combination Therapies

Emerging DDR‐targeted agents, including inhibitors of ATR, DNA‐PK, and CHK1/2, offer new opportunities for rational combination strategies in MM [31, 32]. ATR inhibitors disrupt replication stress responses and sensitize MM cells to DNA damage, while simultaneously enhancing tumor immunogenicity. When combined with IMiDs such as lenalidomide, they may synergistically augment both tumor cell killing and immune activation [31, 32]. Similarly, DNA‐PK inhibitors, by blocking the NHEJ repair pathway, significantly potentiate the cytotoxic effects of DNA‐damaging therapies. Specifically, DNA‐PK inhibition has been shown to sensitize MM cells to alkylating agents and proteasome inhibitors, both of which induce DSBs that require functional NHEJ for cellular survival [32]. Furthermore, CHK1/2 inhibitors force damaged cells into mitotic catastrophe by abrogating cell cycle checkpoints, thereby enhancing the antiproliferative effects of conventional agents [32]. Preclinical studies consistently demonstrate strong synergy across these combinations, supporting a mechanism‐driven therapeutic paradigm in which DDR inhibitors serve as potent sensitizers to establish antimyeloma regimens.

## 7. Resistance Mechanisms and Future Perspectives of DDR‐Targeted Therapy

### 7.1. Acquired Resistance to DDR‐Targeted Therapy

Acquired resistance represents a major challenge for DDR‐targeted therapies in MM, predominantly driven by reversion mutations and compensatory activation of bypass pathways [31, 32]. Reversion mutations restore HR functionality, thereby diminishing the efficacy of PARP inhibitors. For instance, ~20% of PARP inhibitor–resistant MM patients harbor BRCA2 reversion mutations, most commonly p. Gln1756Arg, which re‐establish HR proficiency [31]. Similarly, ATM reversion can restore kinase activity, reactivating DDR signaling and promoting survival under therapeutic pressure [32]. Compensatory pathways further contribute to resistance. MM cells can upregulate NHEJ via DNA‐PK overexpression to circumvent HR deficiency, conferring resistance to PARP inhibition [32]. Activation of the PI3K/AKT axis also mitigates sensitivity to ATR inhibitors by promoting cell survival despite DNA damage [32]. Notably, these mechanisms often coexist, producing multilayered resistance, exemplified by MM cells with simultaneous BRCA reversion and DNA‐PK overexpression [31]. Such complexity underscores the need for combinatorial approaches to overcome DDR‐targeted therapy resistance.

### 7.2. AI‐Driven Precision Oncology

AI‐guided precision oncology offers a transformative framework for DDR‐targeted therapy [33–35]. By integrating multiomics data—including genomics, transcriptomics, epigenomics, and ctDNA—deep learning models generate patient‐specific “DDR defect profiles.” These profiles classify MM into subtypes such as HRD, NHEJ‐deficient, or BER‐deficient, enabling tailored therapeutic selection. For instance, HRD subtypes exhibit significantly higher response rates to PARP inhibition (40% vs. 10%) [33]. AI models can also predict resistance, with ctDNA‐based deep learning achieving 85% accuracy in forecasting PARP inhibitor failure [34]. Beyond risk prediction, AI informs the design of rational combination therapies, recommending dual DDR‐targeted regimens or DDR–immunotherapy combinations optimized for a patient′s unique molecular landscape [33]. Furthermore, AI facilitates dynamic monitoring of DDR clone evolution, providing early relapse alerts 3–6 months ahead of clinical progression [34]. Collectively, AI‐driven frameworks integrate molecular vulnerability mapping, resistance prediction, and therapy optimization, offering a blueprint for individualized DDR‐targeted precision medicine in MM.

## 8. Conclusion and Future Perspectives

DDR gene alterations in MM play pivotal roles not only in disease pathogenesis but also in prognosis, risk stratification, and therapeutic decision‐making. Mutations in key DDR genes, including TP53, ATM, and BRCA2, are associated with significantly shorter progression‐free and OS, and their integration into the R‐ISS or multiomics‐based prognostic models can enhance individualized risk prediction. Liquid biopsy and dynamic monitoring of ctDNA further enable noninvasive tracking of DDR clone evolution and MRD, providing early warning of relapse and real‐time assessment of treatment response. Therapeutically, DDR‐targeted strategies—including synthetic lethality approaches with PARP inhibitors, emerging DDR inhibitors, and combination therapies with conventional antimyeloma agents or IMiDs—offer strong mechanistic rationale for precision treatment. However, acquired resistance mechanisms, such as BRCA/ATM reversion mutations and compensatory repair pathway activation, pose challenges to single‐agent strategies. Next‐generation approaches, including dual‐target DDR inhibitors, sequential therapeutic interventions, and AI‐guided precision medicine, show promise in overcoming resistance and tailoring treatments to individual patients. In summary, DDR alterations provide critical insights for risk assessment and therapeutic guidance in MM, laying the foundation for precise, dynamic, and multidimensional treatment strategies. The integration of multiomics profiling, liquid biopsy–based monitoring, and AI‐assisted clinical decisions holds the potential to achieve fully personalized management of MM, ultimately improving patient outcomes and successfully advancing DDR‐targeted therapy into routine clinical practice.

## Author Contributions

Qian Shen: original draft, visualization, and validation. Ying Wang: investigation, formal analysis, and conceptualization. Liuhuan Cai: visualization. Juan Qian: writing—review and editing, supervision, resources, and funding acquisition. Qian Shen and Ying Wang contributed equally to this work.

## Funding

This research was financially supported by Nantong Natural Science Foundation and Social Livelihood Science and Technology Program Projects (JCZ2023019).

## Disclosure

All authors have read and approved the final manuscript.

## Ethics Statement

The authors have nothing to report.

## Conflicts of Interest

The authors declare no conflicts of interest.

## Data Availability

The data that support the findings of this study are available from the corresponding author upon reasonable request.
